# Photon-counting CT for bullet material differentiation: applications in forensic radiology

**DOI:** 10.1186/s41747-025-00586-x

**Published:** 2025-05-04

**Authors:** Benedikt M. Schaarschmidt, Jan Hegmanns, Jörg Wulff, Viktor Haase, Sebastian Faby, Felix Baum, Christian Bäumer, Sebastian Zensen, Johannes Haubold, Benno Hartung

**Affiliations:** 1 Institute of Diagnostic and Interventional Radiology and Neuroradiology, University Hospital Essen, Essen, Germany; 2 West German Proton Therapy Centre Essen (WPE), Essen, Germany; 3 West German Cancer Centre (WTZ), University Hospital Essen, Essen, Germany; 4 Computed Tomography, Siemens Healthineers AG, Forchheim, Germany; 5 State Criminal Office North Rhine-Westphalia, Düsseldorf, Germany; 6 German Cancer Consortium (DKTK), Essen, Germany; 7 Department of Physics, TU Dortmund University, Dortmund, Germany; 8 Institute of Legal Medicine, University Hospital Essen, Essen, Germany

**Keywords:** Brass, Forensic ballistics, Lead, Tomography (x-ray computed), Wounds (gunshot)

## Abstract

**Background:**

Gunshot deaths due to homicide or military encounters are a major health concern. Noninvasive bullet characterization is of major importance for patients with lodged bullets or in mass disasters with multiple cadavers, which must be prioritized for autopsy. Therefore, the aim of this study was to investigate whether brass and lead bullets can be differentiated using photon-counting CT (PCCT).

**Methods:**

Nine different lead (*n* = 6) or brass (*n* = 3) bullets were investigated on a state-of-the-art PCCT using a clinically unavailable research mode. Here, four image sets were reconstructed for different energy thresholds (20, 55, 72, 90 keV). Three circular regions of interest were placed on the 20-keV threshold images by two readers and automatically copied to the three other threshold images. Based on measured HU mean and max values, dual-energy indices (DEI) were calculated for the low/high energy threshold pairs of 20/90, 55/90, and 72/90 keV.

**Results:**

Significant differences of DEIs between lead and brass projectiles were observed for the 20/90 keV DEI for HU mean ± standard deviation values (Qr40 kernel, lead: -0.085 ± 0.021, brass: 0.024 ± 0.048) and HU max values (Qr40 kernel, lead: -0.093 ± 0.011, brass: 0.023 ± 0.057) (*p* < 0.001 for both). Differences decreased for the 55/90 and 72/90 keV DEIs between the two projectile materials but remained statistically significant.

**Conclusion:**

In this PCCT phantom study, significant differences were observed between lead and brass bullets in the different energy threshold images.

**Relevance statement:**

Photon-counting CT could be a promising tool for bullet identification as significant differences were found in the different energy threshold images for lead and brass bullets, with application in clinical and forensic radiology.

**Key Points:**

- In emergency settings, noninvasive bullet characterization is of importance for law enforcement.
- Bullet material characterization can be performed using photon-counting CT.
- These characteristics can be quantified in the four different energy threshold images.

**Graphical Abstract:**

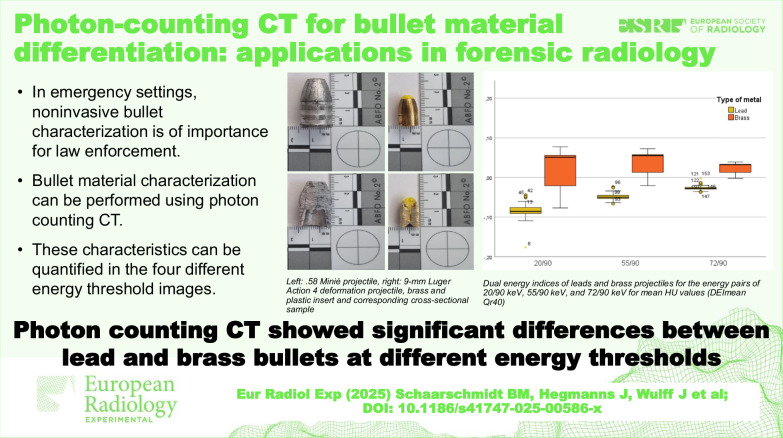

## Background

With an estimated number of 251,000 gunshot deaths worldwide in 2016 and 47,286 estimated gunshot deaths in the USA alone in 2021, gun violence and its medical sequelae are a major healthcare problem [1, 2]. As the majority of firearm deaths are caused by homicide [1], the identification of lodged bullets is of major interest for law enforcement. Furthermore, recent research has demonstrated the advantages of conservative gunshot wound management over surgical projectile removal in various scenarios [3–5]. Therefore, noninvasive bullet characterization in x-ray and computed tomography (CT) examinations is of major importance to narrow down the number of possible firearms used in the shooting and exclude or verify the use of different weapons.

Various morphometric approaches have been proposed, including the visual appearance of the bullet, its shape and caliber measurements [6, 7]. Although modern metal artifact reduction techniques, such as iterative metal artifact reduction [8–10] as well as extended 16-bit CT grayscales [11, 12] improve bullet visualization, their appearance is frequently heavily altered by the impact. This is particularly important for nonmilitary ammunition, such as deformation bullets used by hunters or law enforcement agencies, due to their superior momentary effect on living targets.

In these cases, advanced imaging techniques can be used to differentiate lodged bullets by material decomposition. Although different materials have specific attenuation values, comparing absolute HU is difficult due to photon starvation, beam hardening artifacts [13] and differences attributed to the employed CT scanner type [14]. Repeated CT measurements at different kVp levels or the use of dual-energy CT scanners and the subsequent calculation of so-called dual-energy indices (DEI) as initially proposed by Winklhofer et al [15] might be a promising alternative. To assess HU changes between different energy levels, however, repeated CT scans are necessary. While such examinations are possible in corpses, this approach might be especially problematic in young gunshot victims, where complete bullet removal is not deemed appropriate. Apart from the considerable increase in radiation exposure, patient movement between the repeated CT scans could reduce the reliability of such an examination.

The introduction of novel photon-counting CT (PCCT) scanners into clinical practice might further improve bullet characterization. In contrast to energy-integrating detectors used in previous generations of CT scanners, photon-counting detectors allow for the direct conversion of incoming individual photons into electrical signals without the loss of information concerning their absolute number and energy levels. Therefore, the purpose of the present study was to investigate the potential of PCCT for bullet material differentiation of different projectiles.

## Methods

This preclinical investigation was conducted at the Siemens Healthineers facilities in Forchheim, Germany. As no human or animal subjects were investigated in this study, local ethics committee approval was not required.

### Phantom

**Fig. 1:**
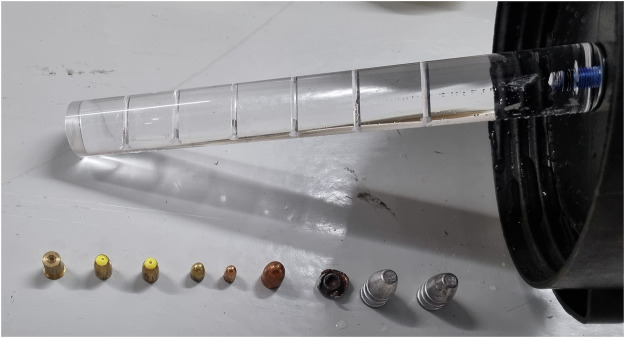
Examined projectiles next to the polymethylmethacrylate rod in the center of the 25-L water container. Bullets examined from left to right: (1–3) 9-mm Luger Action 4 deformation projectiles; (4) 7.65-mm Browning full metal jacket projectile; (5) 6.35-mm Browning full metal jacket projectile; (6) 0.45-inch ACP full metal jacket projectile; (7) 0.22-inch lead round nose high velocity projectile; (8 and 9) 0.58-inch Minié projectiles. ACP, Automatic Colt Pistol

**Fig. 2:**
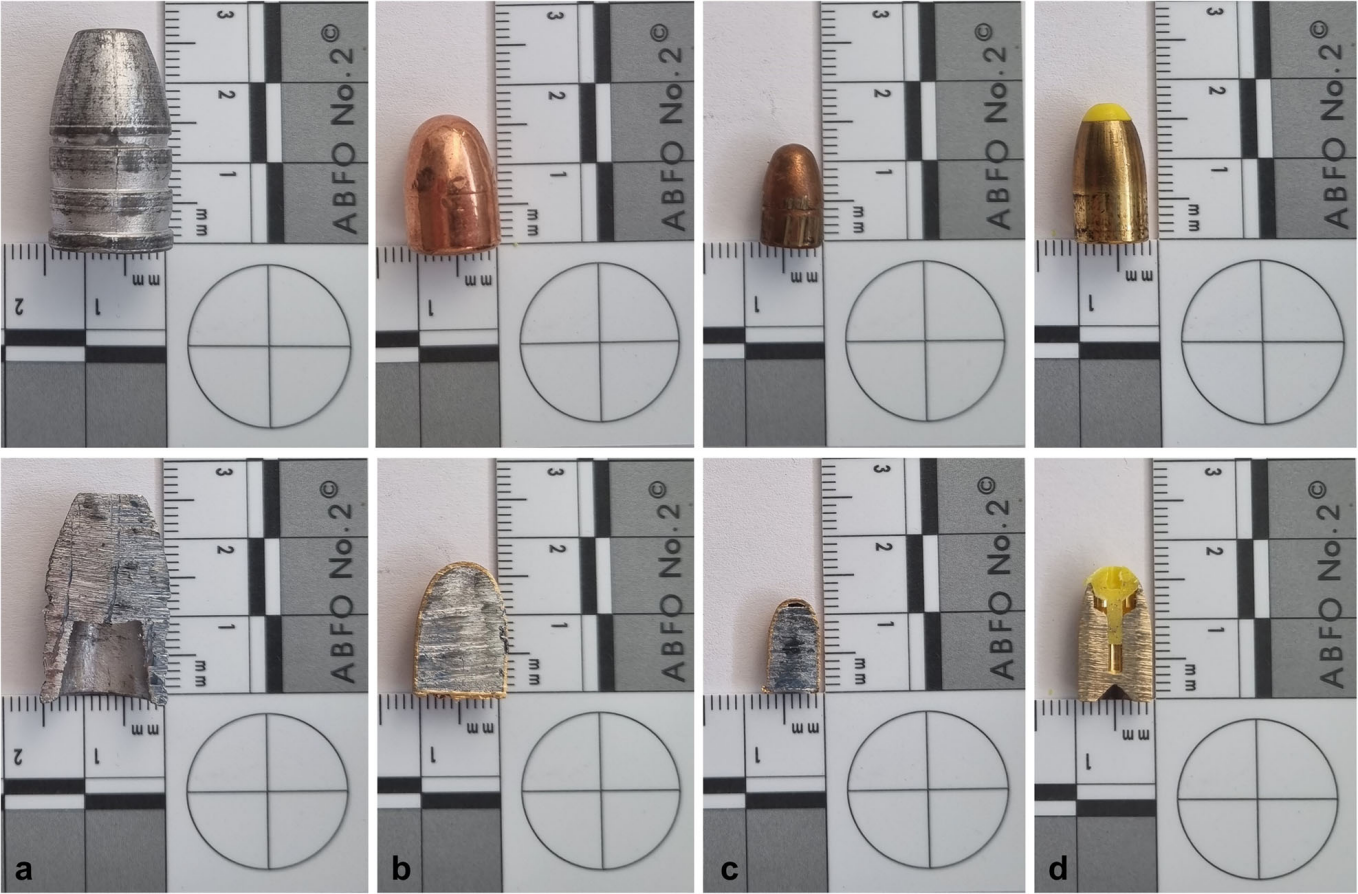
Four exemplary projectiles (upper row) and corresponding cross-sectional sample after mechanical separation into its two halves. **a** 0.58-inch Minié projectile, lead; **b** 0.45-inch Automatic Colt Pistol full metal jacket projectile, tombac coating and lead core; **c** 6.35-mm Browning full metal jacket projectile, tombac coating and lead core; **d** 9-mm Luger Action 4 deformation projectile, brass and plastic insert

A phantom was constructed using a 25-L container with a diameter of 32 cm to mimic the extent of an adult abdomen. A rod made of polymethylmethacrylate was attached to the center of the lid for easy removal of the analyzed objects. Nine projectiles from four different groups were attached to the rod for analysis in the water-filled container (Figs. 1 and 2):

- Group 1, lead, 0.58-inch Minié projectiles (*n* = 2);
- Group 2, lead fragment, 0.22-inch lead round nose high velocity projectile (*n* = 1);
- Group 3, tombac (a brass alloy) coating/lead core:
  - 7.65-mm Browning full metal jacket projectile (*n* = 1);
  - 6.35-mm Browning full metal jacket projectile (*n* = 1);
  - 0.45-inch Automatic Colt Pistol full metal jacket projectile (*n* = 1);
- Group 4, brass (copper/zinc), 9-mm Luger Action 4 deformation projectiles (*n* = 3).

### Data acquisition and image reconstruction

All CT measurements were performed on a NAEOTOM Alpha (software version VB10A, Siemens Healthineers, Forchheim, Germany), a state-of-the-art PCCT with an active detection layer consisting of a cadmium telluride crystal. A new, clinically unavailable research mode allows the output of four reconstructed images of different energy thresholds of the photon-counting detectors, here referred to as threshold images. A threshold image for a given threshold of, *e.g*., 55 keV, is reconstructed from all counted photons in the energy range starting from 55 keV to the highest energy of the x-ray spectrum. This should not be mistaken for a virtual monoenergetic image at a specific, calculated single energy [16]. Currently, there are only a limited number of threshold settings available. One option is the following combination of thresholds: 20, 55, 72, 90 keV. As this new acquisition mode is currently only used for scientific purposes, no preprocessing correction steps are performed on the data prior to image reconstruction, *e.g*., no beam hardening correction.

**Fig. 3:**
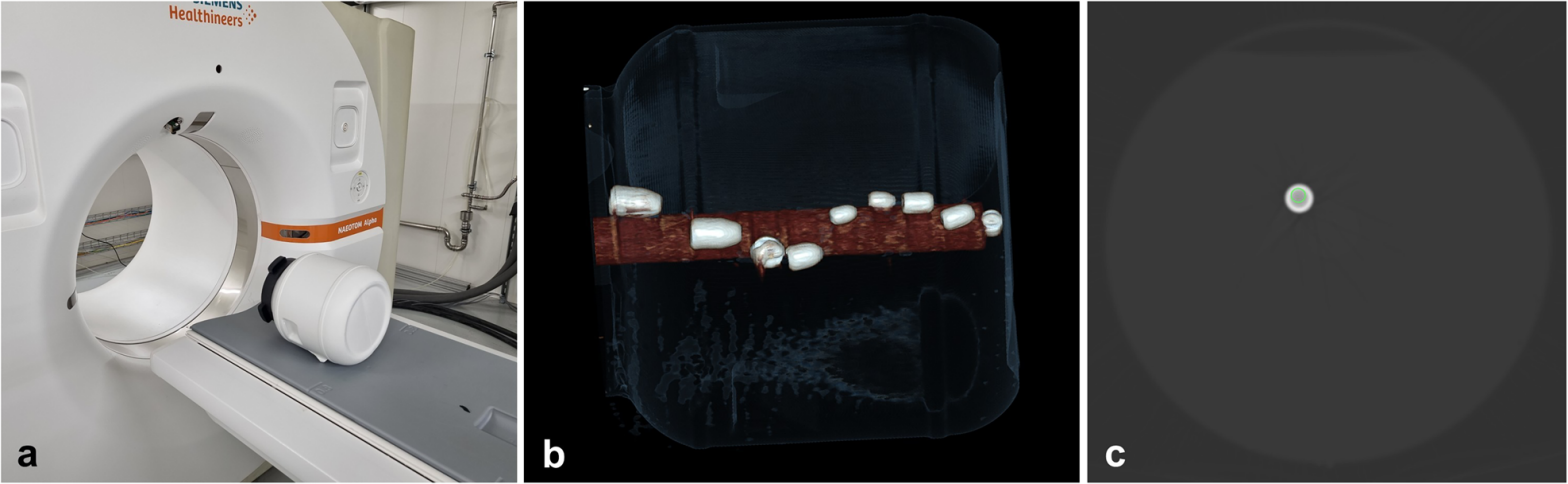
Experimental setup of the present analysis. **a** Position of the phantom on the examination table of the photon-counting CT scanner; **b** volume rendering technique image of the phantom depicting the nine bullets around the central polymethylmethacrylate rod; **c** region-of-interest placement in a lead 0.58-inch Minié projectile

For this experiment, the phantom was scanned in prone position (Fig. 3) in the clinically available standard mode (120 kV, 87 mAs, ultrahigh resolution mode, pitch: 0.85, rotation time: 0.5 s) and in the 4-threshold research mode (140 kV, 87 mAs, pitch 0.8, rotation time 0.25 s). To provide optimal differentiation between the two different projectile cores and their unique K-edge (copper, 8.98 keV; lead, 88.00 keV [17]), the abovementioned threshold option was chosen.

All images were reconstructed using a soft tissue kernel (Qr40) and bone quantitative kernel (Qr72) with a matrix size of 512 × 512, a slice thickness of 3 mm and an increment of 1.5 mm. All images were reconstructed using a 16-bit grayscale providing HU values from -8,192 HU to +57,343 HU.

### Image analysis

All measurements were performed using a dedicated OsiriX workstation (Pixmeo SARL, Bernex, Switzerland). On the 20-keV threshold images reconstructed using the Qr40 kernel, a total of three circular regions of interest (ROIs) were placed by two individual readers (J.H. and B.S.) over the entire bullet volume, avoiding hollows of non-metallic areas of each bullet.

To minimize the effect of beam hardening artifacts, the oval ROIs encompassed the whole center of the bullet avoiding the rims of the bullet. For Action 4 bullets, ROIs were placed laterally of the center to avoid the central plastic insert. Again, the ROI area was maximized to cover as much of the bullet as possible.

Then, the ROIs were automatically copied onto the 55, 72, and 90 keV Qr40 threshold images, the 20, 55, 72, and 90 keV Qr72 threshold images as well as the polychromatic T3D Qr40 and Qr72 images. Mean and maximum HU were recorded for each measurement.

Based on measured HU values (*x*), the dual-energy index (DEI) was calculated for the high/low energy threshold pairs of 20/90 keV, 55/90 keV, and 72/90 keV for mean HU (DEI_mean_) and maximum HU values (DEI_max_) as proposed by Gascho et al [18]:

$$\frac{{x}_{{low}}-{x}_{{high}}}{({x}_{{low}}+{x}_{{high}}+2000)}={DEI}$$

### Statistical analysis

Statistical analysis was performed using SPSS 29.0 (IBM Corp., Chicago, IL, USA). Bullets were divided into two groups according to their core material. For descriptive analysis, mean and standard deviation were calculated for ROI size, HU mean well as for DEI_mean_ and DEI_max_. In accordance with Ruxton et al [19], the two different groups were compared using the unequal variance *t*-test. To avoid α error accumulation, Bonferroni correction was applied. Therefore, *p* < 0.002 indicated statistical significance.

## Results

**Fig. 4:**
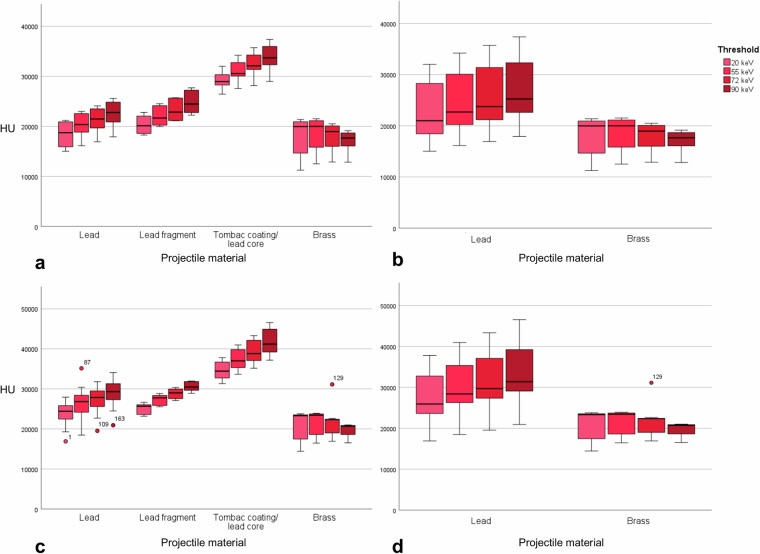
Box plots depicting HU from region-of-interest-based measurements of various projectile groups for HU mean (**a**, **b**) and HU max (**c**, **d**) in the Qr40 images

The mean cross-sectional area for HU measurements was 34.3 ± 16.5 mm² for lead and 12.3 ± 7.5 mm² for brass projectiles. In standard mode, mean HU values were 23,012.5 ± 5,712.3 HU for lead and 20,584.5 ± 3,170.1 HU for brass projectiles in the Qr40 kernel reconstruction and 23,517.9 ± 6,823.6 HU for lead and 21,803.3 ± 3,260.2 HU for brass projectiles in the Qr72 kernel reconstruction. Despite small differences between the two projectile groups, no significant differences were observed for the Qr40 (*p* = 0.050) and the Qr72 kernel (*p* = 0.217). In the 4-threshold research mode, a slight increase in mean HU values could be observed for the four energy threshold levels from 20 to 90 keV in lead projectiles. In brass projectiles, however, a slight rise in mean HU could be observed from the 20 to the 55 keV energy threshold level, followed by a gradual decrease of mean HU in the 72 and 90 keV images. In all energy threshold levels, differences between both projectile types were statistically significant according to the unequal variance *t*-test (Fig. 4, Table 1).

**Table 1:** Hounsfield unit measurements of lead and brass bullets in various CT image reconstructions

Reconstruction kernel	Threshold level	Lead (mean ± SD)	Brass (mean ± SD)	*p*-values
Qr40 (standard)		23,012.5 ± 5712.3	20,584.5 ± 3170.1	0.050
Qr72 (standard)		23,517.9 ± 6823.6	21,803.3 ± 3260.2	0.217
Qr40	20 keV	22,365.5 ± 2543.5	18,234.7 ± 3415.7	0.001^a^
	55 keV	24,103.0 ± 5381.1	18,714.7 ± 2916.4	< 0.001^a^
	72 keV	25,241.6 ± 5596.2	18,082.2 ± 2391.6	< 0.001^a^
	90 keV	26,634.2 ± 5717.5	17,113.5 ± 1929.6	< 0.001^a^
Qr72	20 keV	23,052.9 ± 6093.9	18,889.5 ± 3504.1	0.003^a^
	55 keV	24,503.8 ± 6384.2	19,425.9 ± 2965.0	< 0.001^a^
	72 keV	25,690.2 ± 6604.8	18,660.2 ± 2351.6	< 0.001^a^
	90 keV	27,211.8 ± 6865.2	17,778.6 ± 1888.1	< 0.001^a^

*SD* Standard deviation

^a^ Statistical significance

**Fig. 5:**
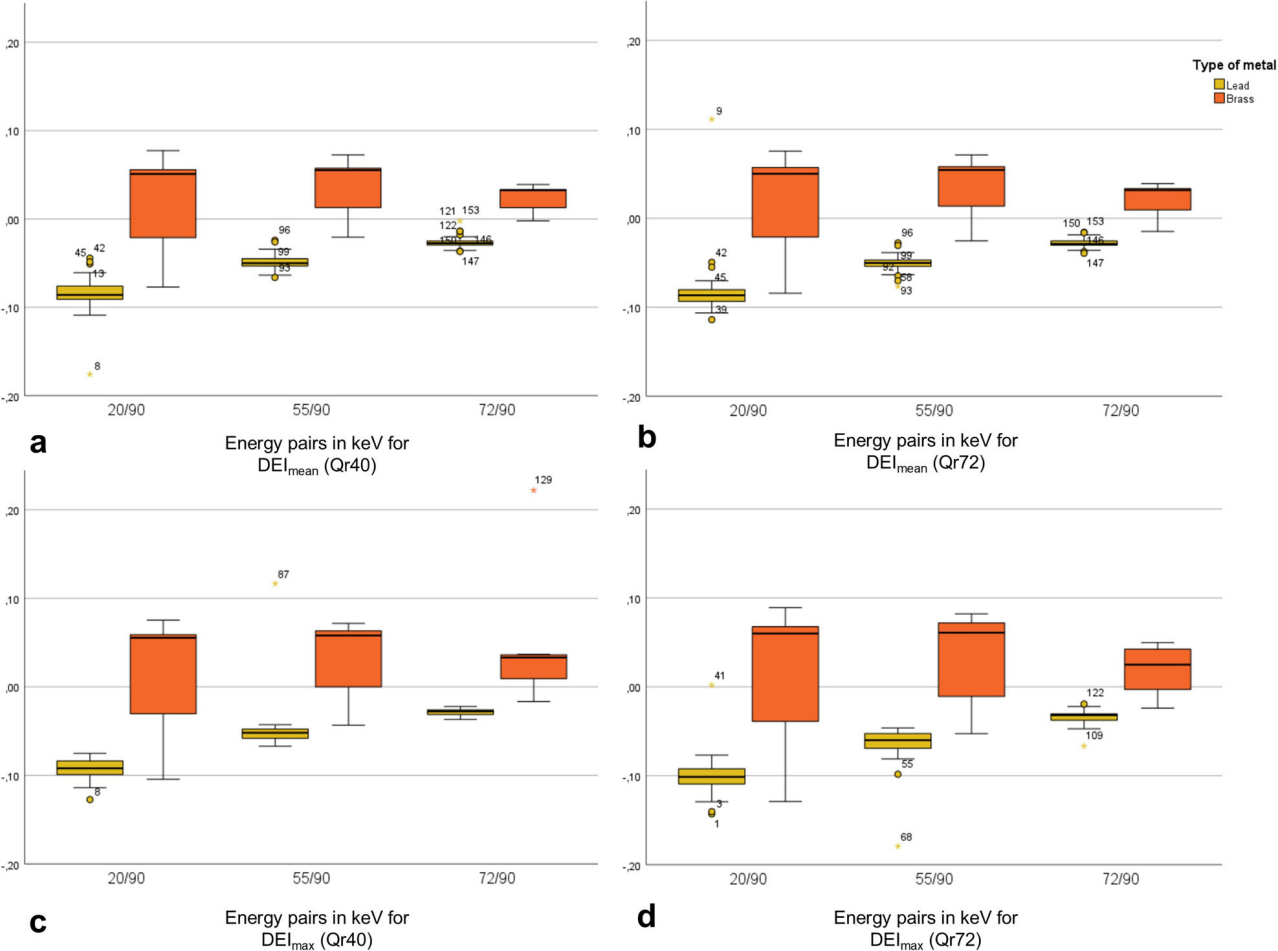
Box plots depicting dual-energy indices of leads and brass projectiles. Based on region-of-interest-based measurements, DEIs were calculated for the energy pairs of 20/90 keV, 55/90 keV, and 72/90 keV for mean HU (**a** DEI_mean_ Qr40; **b** DEI_mean_ Qr72) and maximum HU values (**c** DEI_max_ Qr40; **d** DEI_max_ Qr72). DEI, Dual-energy index; SD, Standard deviation

In accordance with the literature, DEIs were calculated for mean and maximum HU and compared between lead and brass bullets. As in the sole HU comparisons, we observed significant differences in DEIs between the two projectile types for 20/90 keV for HU mean (Qr40 kernel, lead -0.085 ± 0.021, brass 0.024 ± 0.048, *p* < 0.001; Qr72 kernel, lead: -0.081 ± 0.035, brass 0.023 ± 0.050, *p* < 0.001) and HU max (Qr40 kernel, lead -0.093 ± 0.011, brass 0.023 ± 0.057, *p* < 0.001; Qr72 kernel, lead -0.101 ± 0.023, brass 0.023 ± 0.067, *p* < 0.001), irrespective of the chosen reconstruction kernel. Although the differences in the DEIs for 55/90 keV and 72/90 keV decreased, the differences between the different projectile groups remained statistically significant (Fig. 5, Table 2).

**Table 2:** DEI_mean_ and DEI_max_ values for lead and brass bullets in various CT image reconstructions

Type of DEI index	Threshold levels	Lead (mean ± SD)	Brass (mean ± SD)	*p*-values
Qr40 DEI_mean_	20/90 keV	-0.085 ± 0.021	0.024 ± 0.048	< 0.001^a^
	55/90 keV	-0.049 ± 0.009	0.039 ± 0.029	< 0.001^a^
	72/90 keV	-0.026 ± 0.007	0.025 ± 0.013	< 0.001^a^
Qr40 DEI_max_	20/90 keV	-0.093 ± 0.011	0.023 ± 0.057	< 0.001^a^
	55/90 keV	-0.048 ± 0.029	0.037 ± 0.037	< 0.001^a^
	72/90 keV	-0.029 ± 0.004	0.034 ± 0.050	< 0.001^a^
Qr72 DEI_mean_	20/90 keV	-0.081 ± 0.035	0.023 ± 0.050	< 0.001^a^
	55/90 keV	-0.051 ± 0.010	0.039 ± 0.030	< 0.001^a^
	72/90 keV	-0.028 ± 0.005	0.022 ± 0.016	< 0.001^a^
Qr72 DEI_max_	20/90 keV	-0.101 ± 0.023	0.023 ± 0.067	< 0.001^a^
	55/90 keV	-0.065 ± 0.023	0.038 ± 0.045	< 0.001^a^
	72/90 keV	-0.034 ± 0.009	0.019 ± 0.028	< 0.001^a^

*DEI* Dual-energy index, *SD* Standard deviation

^a^ Statistical significance

## Discussion

The correct identification of bullets is of major importance for law enforcement agencies worldwide. As new treatment regimens favor conservative management of gunshot wounds over complete projectile removal, noninvasive characterization of lodged bullets is crucial. The present study yielded the following results: in the standard scan mode, no significant differences in HU were detected between lead and brass bullets for two different reconstruction kernels; however, specific changes could be observed in the four different threshold images obtained by PCCT for lead and brass bullets. These specific changes could be quantified successfully using DEIs. Here, significant differences for DEIs between lead and brass bullets could be observed.

CT imaging is an excellent modality to detect lodged bullets in the human body and to identify accompanying injuries [20]. In addition to clinically indicated CT examinations, the increased use of postmortem CT examinations in forensic examinations has further increased the interest to image bullets as well as shooting trajectories in CT [21]. In recent CT scanners, bullet visualization has further improved by new metal artifact reduction techniques such as iterative image reconstruction [9, 10, 22]. Although morphometric bullet identification is possible, as demonstrated by Gascho et al, various bullet types demand experience in this field as well as the availability of reference tables [7, 23]. Additionally, bullet appearance can be heavily altered by the impact, further complicating this approach. To overcome these limitations, bullet differentiation by noninvasive by material identification might be a promising, alternative approach. Paulis et al attempted to use extended 16-bit CT scales with a maximum HU value of +30,710 instead of +3,095 HU to correctly quantify the attenuation of dislodged bullets [23]. However, these attempts were futile for two main reasons. First, the attenuation of most projectiles, especially in extremely dense materials such as lead, causes central photon starvation artifacts as well as beam hardening artifacts at the outer rim of the projectiles. Second, many materials used for bullet manufacturing, such as iron, copper and zinc, have similar atomic numbers that cannot be differentiated by a sole single energy CT examination [23]. Third, these measurements can be impaired by the purity of the examined objects, especially in the presence of metallic alloys [13].

The examination of a single object with different energy levels by dual-energy CT and the quantification of attenuation differences by DEIs is already used in clinical routine, *e.g*., for the differentiation of urinary stones [24]. Here, various researchers demonstrated that this technique can be used to differentiate various materials [25], brass and lead bullets [18, 26], as well as ferromagnetic and nonferromagnetic bullets [15] in CT. However, the analysis is dependent on the energy levels chosen at the beginning of the examination process. Furthermore, the available energy levels are far higher than the characteristic K-edge of most metals used for bullet manufacturing, especially copper, zinc, and iron. To overcome such limitations, the recently introduced PCCT scanners could be a promising alternative.

Most currently available CT scanners rely on energy-integrating detectors. Here, incoming photons are converted into light using solid-state scintillators, which is detected by photodiodes. During this process, however, information about the number of incoming photons and their individual energy levels is lost. In photon-counting detectors, each photon is detected individually, and its energy is recorded. In the present study, we demonstrated the feasibility of PCCT to differentiate successfully between brass and lead bullets in a phantom study. Here, we were able to show that HU values developed differently between the four different energy level images. In accordance with prior research by Gascho et al [15] and Winklhofer et al [26], the DEIs calculated between the various HU values showed significant differences between the two bullet types. Therefore, PCCT could be a new option for noninvasive bullet characterization: either in patients with lodged bullets or in mass disasters with multiple cadavers demanding prioritization for autopsy. However, further investigation of actual bullet fragments in organic tissue is necessary to substantiate these findings.

The present study has some limitations. First, beam hardening correction is not yet implemented in the 4-threshold research mode. As beam hardening is a major problem in the characterization of foreign bodies, this fact might impair correct HU measurement. Second, our analysis used a simplified water phantom with similar bullet orientation and the analysis of only one fragmented bullet for an initial analysis. Here, further examinations employing anthropomorphic phantoms or animal cadavers might be useful. Third, the analysis was based on a selected set of brass and lead bullets. Especially ferromagnetic projectiles, which are of high clinical relevance due to the ongoing conflicts in the Middle East and Ukraine, were not analyzed in this study. Here, PCCT may be useful to identify patients with lodged, ferromagnetic bullets, which should not undergo MRI examinations due to bullet movement [27]. Further analysis is warranted once the 4-threshold research mode is available for regular use.

In conclusion, the present phantom study demonstrates the potential of PCCT for noninvasive analysis of lodged bullets. As significant differences between lead and brass bullets were observed, PCCT could be a promising tool for bullet identification in both clinical and forensic radiology.

## Data Availability

The datasets generated and/or analyzed during the current study are not publicly available due to continuous research on this topic, but are available from the corresponding author upon reasonable request.

## Abbreviations

CT: Computed tomography
DEI: Dual-energy index
PCCT: Photon-counting CT
ROI: Region of interest
